# Acute glycaemic effects of co-trimoxazole at prophylactic dose in healthy adults

**DOI:** 10.1186/s12902-016-0142-6

**Published:** 2016-11-15

**Authors:** Bernold Kenteu, Jean Jacques N. Noubiap, Martine Claude Etoa, Marcel Azabji-Kenfack, Mesmin Dehayem, Eugene Sobngwi

**Affiliations:** 1 Department of Internal Medicine and Specialties, Faculty of Medicine and Biomedical Sciences, University of Yaoundé 1, Yaoundé, Cameroon; 2 Department of Medicine, Groote Schuur Hospital and University of Cape Town, Cape Town, South Africa; 3 National Obesity Centre, Diabetes and Metabolic Diseases Unit, Yaoundé Central Hospital, Yaoundé, Cameroon; 4 Department of Physiological Sciences and Biochemistry, Faculty of Medicine and Biomedical Sciences, University of Yaoundé 1, Yaoundé, Cameroon; 5 Biotechnology Centre, University of Yaoundé 1, Yaoundé, Cameroon; 6 National Obesity Center, Yaoundé Central Hospital and Faculty of Medicine and Biomedical Sciences, University of Yaoundé 1, Yaoundé, Cameroon

**Keywords:** Co-trimoxazole, Glycaemia, Adults

## Abstract

**Background:**

Cases of severe hypoglycaemia were reported in HIV/AIDS patients receiving high dose of the sulfonylurea co-trimoxazole for opportunistic infections. Whether co-trimoxazole at prophylactic dose would induce similar side effects is unknown. We aimed to investigate the acute effects of co-trimoxazole at prophylactic dose on glucose metabolism in healthy adults.

**Methods:**

We enrolled 20 healthy volunteers (15 males and 5 females) aged 23.0 (SD 2.0) years, with mean BMI of 22.3 (SD 3.6) Kg/m^2^ with normal glucose tolerance, hepatic and renal function. We performed a 75-g oral glucose tolerance test (OGTT) with and without concomitant oral co-trimoxazole administered 60 min before the test. Blood glucose response was measured using a capillary test at baseline and at 30, 60, 90, 120 and 180 min following oral glucose load on the two occasions. C-peptide response was also measured. Absolute values of blood glucose and C-peptide with and without co-trimoxazole were compared using the Wilcoxon test.

**Results:**

During the OGTT without co-trimoxazole (control) vs. the OGTT with co-trimoxazole (test), the glycaemia varied from 4.83 (SD 0.39) mmol/l vs. 4.72 (SD 0.28) mmol/l at T0 (*P* = 0.667), to 8.00 (SD 1.11) mmol/l vs. 7.44 (SD 0.78) mmol/l at T30 (*P* = 0.048), 8.00 (SD 1.17) mmol/l vs. 7.67 (SD 1.00) mmol/l at T60 (*P* = 0.121), 7.33 (SD 0.94) mmol/l vs. 7.11 (SD 0.83) mmol/l at T90 (*P* = 0.205), 6.78 (SD 1.00) mmol/l vs. 6.67 (SD 1.00) mmol/l at T120 (*P* = 0.351) and 4.72 (SD 1.39) mmol/l vs. 4.72 (SD 1.56) mmol/l at T180 (*P* = 0.747). The ratio of area under the glycaemia curve during the control and test investigation was 96.7 %, thus a 3.3 decreased glycaemic response (*p* = 0.062). A decrease of glycaemia by more than 10 % occurred in 6/20 participants at T30, 7/20 participants at T60 and 1/20 participant at T30 and T60. None of the volunteers experienced co-trimoxazole-induced hypoglycaemia. At the same time, the C-peptide response during the control vs. the test investigation varied from 278.1 (SD 57.5) pmol/l vs. 242.8 (SD 42.5) pmol/l at T0 (*P* = 0.138), to 1845.6 (SD 423.6) pmol/l vs. 2340.6 (SD 701.3) pmol/l at T60 (*P* = 0.345) and 1049.8 (SD 503.1) pmol/l vs. 1041.63 (SD 824.21) pmol/l at T180 (*P* = 0.893).

**Conclusion:**

Ninety minutes after its administration, co-trimoxazole induced a significant reduction of the early glycaemic response to oral glucose in parallel with a 27-% increase in insulin secretory response. Co-trimoxazole induced within 120 min a more than 10-% blood glucose reduction in 2/3 of participants. However none of the volunteers experienced hypoglycaemia.

## Background

The HIV/AIDS is a threatening condition affecting 31 million of persons in the world and which has been responsible for 39 million of deaths since the beginning of the pandemic (UNAIDS 2014). That condition leads to a lowering of the immunity causing a high susceptibility to infections. In HIV/AIDS patients the use of co-trimoxazole for the treatment and prevention of opportunistic infections is recommend and is associated with a significant reduction in morbidity and mortality [1, 2].

Despite the inescapable place of co-trimoxazole in the management of HIV/AIDS patients, its use is not risks free [3]. It is responsible for a wide range of side effects such as nausea, vomiting, allergic skin reactions and hypoglycaemia [3]. Hypoglycaemia is an uncommon complication of co-trimoxazole use, maybe for lack of monitoring but, cases of severe hypoglycaemia were reported in patients receiving high dose of co-trimoxazole for opportunistic infections [4, 5]. Those hypoglycaemias were concomitant to an increase in insulin secretion what suggesting a sulfonylurea-like mechanism. Likewise a study highlighted the hypoglycaemic potential of co-trimoxazole in glipizide and glyburide users [6]. Whether co-trimoxazole at prophylactic dose would induce similar side effects is unknown. The aim of this study was to assess the acute glycaemic effects of co-trimoxazole at prophylactic dose in healthy adults.

## Methods

### Setting and participants

The study was conducted at the National Obesity Center, Yaounde Central Hospital. We enrolled 20 healthy adults, living in Yaounde (Cameroon) during the study period; with no contraindication to co-trimoxazole use and free of any medication for other purpose. Other selection criteria included fasting blood sugar of less than 7 mmol/l; Alanine Aminotransferase (ALAT) of less than tree fold the normal; and a glomerular filtration rate superior to 60 ml/min/1.73 m^2^ of body surface.

### Procedure

Each participant underwent on two separated occasion a 75-g oral glucose tolerance test, with (test) and without (control) oral administration of co-trimoxazole, 60 min before the oral glucose load when indicated. The two OGTT were separated by a period of 48 h and each participant was his own witness. To minimise systematic errors, one half of the participants started with the test OGTT and the other half with the control OGTT. The starting order was determined randomly. The co-trimoxazole used was named BERLOCID, and were manufactured by the MENARINI group in Berlin, Germany. The tablets contained 960 mg of co-trimoxazole. Blood glucose response was measured using a OneTouch Ultra2 glucose meter at baseline and 30, 60, 90, 120 and 180 min following the oral glucose load on the two occasions. The insulin response was also assessed by a measure of C-peptide level at baseline, 60 and 180 min after the oral glucose load on the two occasions. The c-peptide level was determined using the MercodiaUltrasensitive C-peptide ELISA (Mercodia AB, Sylveniusgatan 8A, SE-754 50 Uppsala, Sweden); based on the direct sandwich technique in which two monoclonal antibodies are directed against separate antigenic determinants on the C-peptide molecule. This test has a detection limit of 2.5 pmol/L and provides less than 1.8 % of cross reactions.

### Outcomes

The primary clinical outcome was a variation in blood glucose response of more than 20 %, 2 h after the oral administration of co-trimoxazole during the OGTT with co-trimoxazole comparatively to the blood glucose response during the OGTT without co-trimoxazole. The secondary outcomes considered were a variation of more than 10 % of the blood glucose response, any time after the administration of co-trimoxazole and a variation of the insulin secretory response during the OGTT with co-trimoxazole comparatively to the OGTT without co-trimoxazole.

### Statistical analysis

Data was coded, entered and analyzed using the Statistical Package for Social Science (SPSS) version 21.0 for Windows (IBM Corp. Released 2012. IBM SPSS Statistics for Windows, Version 21.0. Armonk, NY: IBM Corp.). We compared absolute value of blood glucose response and C-peptide secretory response with the non-parametric Wilcoxon test. A *p* value of less than 0.05 was considered statistically significant.

## Results

### Characteristics of the study population

We enrolled 20 volunteers aged 23.0 (SD 2.0), with mean BMI of 22.3 (SD 3.6) kg/m^2^; mean fasting blood glucose of 4.89 (SD 0.33) mmol/l; mean alanine aminotransferase of 5.58 (SD 3.56) ui/l and a mean glomerular filtration rate of 110.5 (SD 32.8) (Table 1).

**Table 1:** Baseline characteristics of the study population

Characteristics	Mean values (SD)
Age	23 (2)
Sex (M/F)	(15/5)
Body mass index (kg/m^2^)	22.3 (3.6)
Systolic bloc pressure (mmHg)	118 (11)
Diastolic blood pressure (mmHg)	75 (10)
Fasting blood glucose (mmol/l)	4.89 (0.33)
Alanine aninotranferase (IU/l)	5.58 (3.56)
Glomerular filtration rate (ml/min/1.73 m^2^)	110.5 (32.8)

*SD* standard deviation

### Variations in the glycaemic response during the control versus the test OGTT

**Fig. 1:**
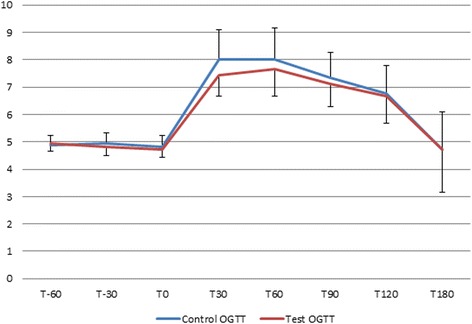
Test versus control OGTT: Compared glycaemic response. Caption: x-axis: time in minutes, y-axis glycemia in mmol/l

During the control OGTT vs. the test OGTT, the glycaemia varied from 4.83(SD 0.39) mmol/l vs. 4.72 (SD 0.28) mmol/l at T0 (*P* = 0.667), to 8.00 (SD 1.11) mmol/l vs. 7.44 (SD 0.78) mmol/l at T30 (*P* = 0.048), 8.00 (SD 1.17) mmol/l vs. 7.67 (SD 1.00) mmol/l at T60 (*P* = 0.121), 7.33 (SD 0.94) mmol/l vs. 7.11 (SD 0.83) mmol/l at T90 (*P* = 0.205), 6.78 (SD 1,00) vs. 6.67 (SD 1.00) mmol/l at T120 (*P* = 0.351) and 4.72 (SD 1.39) mmol/l vs. 4.72 (SD 1.56) mmol/l at T180 (*P* = 0.747) (Table 2). The ratio of area under the glycaemia curve during the control and test investigation was 96.7 %, thus a 3.3 decreased glycaemic response (*p* = 0.062) (Fig. 1). A decrease of glycaemia by more than 10 % occurred in 6/20 participants at T30, 7/20 participants at T60 and 1/20 participant at T30 and T60.

**Table 2:** Variations in the glycaemic response during the control versus the test OGTT

Glycaemia during control OGTT		Glycaemia during Test OGTT		
Time (minutes)	Mean (SD)	Mean (SD)	Difference (%)	*p* value
T-60	4.89 (0.33)	4.94 (0.28)	−1.14	–
T0	4.83 (0.39)	4.72 (0.28)	2.30	0,667
T30	8.00 (1.11)	7.44 (0.78)	6.94	0,048
T60	8.00 (1.17)	7.67 (1.00)	4.17	0,121
T90	7.33 (0.94)	7.11 (0.83)	3.03	0,205
T120	6.78 (1.00)	6.67 (1.00)	1.64	0,351
T180	4.72 (1.39)	4.72 (1.56)	0	0,747

*SD* Standard deviation, Glycaemia are express in mmol/l and mean are compared by the Wilcoxon test

### Variations in the insulin secretory response during the control versus the test OGTT

**Fig. 2:**
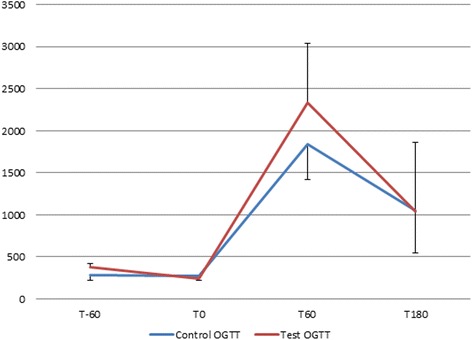
Test versus control OGTT: compared C-peptide secretory response. Caption: x-axis: time in minutes, y-axis c-peptide in pmol/l

The C-peptide response during the control vs. the test investigation varied from 278.1 (SD 57.5) pmol/l vs. 242.8 (SD 42.5) pmol/l at T0 (*P* = 0.138), to 1845.6 (SD 423.6) pmol/l vs. 2340.6 (SD 701.3) pmol/l at T60 (*P* = 0.345) and 1049.8 (SD 503.1) pmol/l vs. 1041.63 (SD 824.21) pmol/l at T180 (*P* = 0.893) (Fig. 2).

### Rate of co-trimoxazole induced hypoglycaemia

None of the participants experienced co-trimoxazole induced hypoglycaemia.

## Discussion

The co-trimoxazole is an antibacterial sulfonamide unavoidable in the management of HIV/AIDS patients but, it sometimes induced severe and even protracted hypoglycaemia when used in high dose. Our study aimed to investigate the acute effects of co-trimoxazole at prophylactic dose on glucose metabolism in healthy adults. We noted an acute reduction in the glycaemic response, with 70 % of the participants experiencing a reduction of more than 10 % in the glycaemic response during the test OGTT compared to the control OGTT, some of them 90 min and others 120 min after co-trimoxazole administration. This reduction in glycaemic response was concomitant to an increase in insulin secretory response by 27 %.

Strevel and al. reported cases of severe and protracted hypoglycaemia secondary to high doses of co-trimoxazole in patients with hypoglycaemia risk factors [4, 5]. Those risk factors include chronic renal failure (93 %), malnutrition, and concomitant administration of propoxyphene. Nunnari and al. also reported cases of hypoglycaemia secondary to high dose of co-trimoxazole. On the other hand, Schelleman and al. highlighted co-trimoxazole induced hypoglycaemia when associated to glipizide or glyburide [6]. The absence of hypoglycaemia in our experimentation could be related to our procedure. First we used a low dose of co-trimoxazole; secondly we used a dynamic test (a 75 g OGTT) which had the advantage to further stimulate the insulin secretion but at the same time prevent hypoglycaemia. Furthermore, those hypoglycaemias occurred in patients with others co-morbidities [4] or taking hypoglycaemic drug [4, 6] compared to our study where volunteers had no co-morbidities and conserved mechanism of counter regulation, which prevent hypoglycaemia and which is probably responsible of the trend to normalization 120 min after co-trimoxazole administration. Indeed, in healthy person, the counter regulatory mechanism are not only activated to reverse hypoglycaemia but, also to prevent its occurrence. That could justify the absence of hypoglycaemia signs. This is correlated by the study of Natalie S. Schwartz and al. which showed that the glycemic thresholds for activation of glucose counter regulatory systems are higher than the threshold for symptoms [7]. Thereby it would be interesting to assess the hypoglycaemic potential of co-trimoxazole in patients with co-morbidities and altered counter regulating mechanism.

Plausible mechanisms by which hypoglycaemia might occur is the stimulation of insulin secretion as reported by Nunnari and al. and Strevel and al. [4, 5], suggesting a sulfonylurea-like mechanism. So co-trimoxazole would fixed itself on the SUR1 subunit of the beta-cell ATP-sensitive potassium channel leading to the closure of ATP-sensitive potassium channels, then to the depolarization of the cell membrane with the opening of voltage-gated calcium channels. This produces an influx of calcium that stimulates fusion of the docked insulin-containing vesicles with the cell membrane and secretion of insulin into the extracellular fluid by exocytosis. This is reinforced by the history of the discovery of sulfonylurea. Indeed, Marcel Janbon reported cases of reversible coma after glucose administration in patients treated with the antibacterial sulphonamide 2254 RP for typhoid fever. This finding aroused interrogations and this is how A. Loubatières highlighted the hypoglycaemic potential of 2254 RP.

This study shows that at prophylactic dose co-trimoxazole can be safely administered in healthy adult, and this is because of the activation of the counter regulatory system. Nevertheless a special attention should be paid when co-trimoxazole is administered at high dose like in the treatment of Pneumocystis jiroveci pneumonia, and to patients with comorbidities including renal insufficiency or malnutrition. Indeed malnutrition and kidney dysfunction are risks factors for severe hypoglycaemia in patients using high dose of co-trimoxazole as reported by Strevel and al. in a case series [4]. Moreover, Hemkisoy and al. and Arem and al. reported a case of co-trimoxazole-induced hypoglycaemia in a malnourish patient and in another one with in chronic renal failure [8, 9]. A monitoring of blood glucose level is advised in case of concomitant administration of co-trimoxazole and sulfonylureas, in order to rapidly detect and treat a potential hypoglycaemia due an excessive insulin secretion, especially in patients with relevant comorbidities.

## Conclusion

Our study shows that 960 mg of co-trimoxazole reduce the glycaemic response within 90 to 120 min after his administration but no hypoglycaemia was observed (probably related to the procedure). This reduction in glycaemic response was associated with an increase in insulin secretory response. Although ours finding cannot prove nor exclude a co-trimoxazole induced hypoglycaemia at prophylactic dose, we suggest to clinicians to be aware of a hypoglycaemic potential of co-trimoxazole and thus increase alertness, especially in patients receiving high dose, with comorbidities or polymedicated patients.

## Abbreviations

OGTT: 75-g oral glucose tolerance test
SD: Standard deviation
